# Melatonin Regulates the Proliferation of Liaoning Cashmere Goat Skin Fibroblasts via the lncRNA16913.1-chi-miR-195-5p-FZD6 Axis

**DOI:** 10.3390/ani16152420

**Published:** 2026-08-05

**Authors:** Weiyu Fan, Linlin Cong, Na Bao, Xin Wang, Yaning Ge, Jiaxin Song, Mei Jin

**Affiliations:** School of Life Science, Liaoning Normal University, Dalian 116081, China; fwy104828@163.com (W.F.); x2085613092@163.com (L.C.); 15942625795@163.com (N.B.); 19587906891@163.com (X.W.); a13795180801@163.com (Y.G.); sjxxin0422@163.com (J.S.)

**Keywords:** Liaoning cashmere goat, LncRNA16913.1, chi-miR-195-5p, FZD6, skin fibroblast

## Abstract

Liaoning cashmere goat is a unique Chinese cashmere breed, and skin fibroblast proliferation determines hair follicle development and cashmere quality. Melatonin regulates cutaneous cell activity, and lncRNAs function as ceRNA to sponge miRNAs and modulate target genes. Whether melatonin controls fibroblast proliferation through an lncRNA-miRNA-mRNA cascade remains unclear. This study explored how melatonin mediates the LncRNA16913.1-chi-miR-195-5p-FZD6 axis to regulate cashmere goat skin fibroblast proliferation. We constructed gain-of-function and loss-of-function models of LncRNA16913.1 and chi-miR-195-5p. Immunohistochemistry located FZD6 in hair follicles; RT-qPCR, Western blot, CCK-8, EdU and flow cytometry detected gene expression and cell phenotypes. Bioinformatic prediction, dual-luciferase assay, fluorescence in situ hybridization, RNA pull-down and rescue tests verified molecular binding relationships. Melatonin upregulated LncRNA16913.1 and FZD6, yet downregulated chi-miR-195-5p, with FZD6 mainly distributed in hair follicle inner and outer root sheaths. Inhibiting chi-miR-195-5p boosted fibroblast proliferation, restrained apoptosis and accelerated the cell cycle. chi-miR-195-5p directly suppressed FZD6, while cytoplasmic LncRNA16913.1 sequestered this miRNA to recover FZD6 expression. Silencing LncRNA16913.1 eliminated melatonin-mediated FZD6 upregulation. In short, melatonin increased LncRNA16913.1, which sequestered chi-miR-195-5p to release FZD6 and facilitate fibro-blast proliferation. Combined dual-luciferase, bidirectional rescue and cellular phenotypic assays collectively verified the fact that FZD6 acts as the functional downstream target of chi-miR-195-5p to mediate fibroblast proliferation in vitro.

## Simple Summary

This study explored whether melatonin-regulated lncRNA16913.1, chi-miR-195-5p and FZD6 interact with each other to co-regulate the proliferation of Liaoning cashmere goat skin fibroblasts. Overexpression and interference models of lncRNA16913.1 and chi-miR-195-5p were constructed, followed by tissue staining, cell proliferation assays and a series of molecular binding verification experiments. The results revealed that chi-miR-195-5p inhibits cell proliferation, while lncRNA16913.1 acts as a ceRNA to sponge chi-miR-195-5p and thereby upregulate FZD6 expression. This study fully elucidates a melatonin-mediated molecular regulatory pathway, and provides in vitro theoretical reference and candidate molecular targets for subsequent in vivo validation and follow-up cashmere goat breeding research.

## 1. Introduction

Cashmere growth is modulated by diverse endogenous and exogenous regulators, among which melatonin (MT) dominates cashmere development via remodeling non-coding RNA (ncRNA) expression profiles. Major ncRNA subtypes include lncRNAs, miRNAs and circRNAs; lncRNA can act as competing endogenous RNA (ceRNA) to sequester miRNAs and de-repress downstream target genes, and ceRNA networks have become a research hotspot in cashmere follicle regulation [1]. Hu et al. reported that miR-143 targets CUX1 and inhibits dermal cell proliferation, thereby affecting hair follicle development [2]. Lu et al’s studies have found that injectable platelet-rich fibrin promotes hair follicle regeneration by accelerating the proliferation of human dermal cells [3]. Collectively, these studies have demonstrated that the proliferation of skin fibroblasts is closely correlated with hair follicle development. Accordingly, skin fibroblasts were selected as the research subject for our further mechanistic investigation.

Frizzled 6 (FZD6), an essential transmembrane receptor of the Wnt pathway, governs mammalian hair follicle patterning, polarity and cyclic progression [4,5,6]. Fzd6 knockout mice display severe disorder of follicle orientation, whereas FZD6 expression and function remain uncharacterized in Liaoning cashmere goat skin follicles [7,8,9]. MiR-195-5p, a conserved follicle-enriched miRNA, acts as a negative modulator of hair follicle cycling with an expression pattern opposite to FZD6. Existing studies have validated the fact that miR-195-5p directly targets the 3′UTR of FZD6 to suppress its transcription in tumor cells, yet this targeting interaction has not been verified in Liaoning cashmere goat skin fibroblasts [10,11,12]. Recent transcriptomic studies across multiple cashmere goat breeds have identified numerous lncRNA-miRNA-mRNA ceRNA axes participating in follicle development and cashmere trait formation, confirming the universal regulatory role of ceRNA networks during cashmere growth [13,14,15,16,17,18,19].

In our previous research, whole-transcriptome sequencing was performed on skin fibroblasts from Liaoning cashmere goats with or without 200 ng/L melatonin treatment. A novel melatonin-responsive lncRNA designated MSTRG.16913.1 was identified, which was renamed LncRNA16913.1 in the present study [20]. Based on these transcriptomic data, we constructed a ceRNA regulatory axis consisting of LncRNA16913.1, chi-miR-195-5p and FZD6. We hypothesized that LncRNA16913.1 might act as a ceRNA to sponge chi-miR-195-5p, thereby modulating FZD6 expression and mediating the biological effects of melatonin on cashmere goat skin fibroblasts. To verify this hypothesis, the current study was designed to achieve the following objectives: (1) to investigate the regulatory effects of melatonin on the expression of LncRNA16913.1, chi-miR-195-5p and FZD6; (2) to clarify the regulatory effect of chi-miR-195-5p on the proliferation of skin fibroblasts derived from Liaoning cashmere goats; (3) to validate the molecular sponge interaction between LncRNA16913.1 and chi-miR-195-5p; and (4) to clarify whether LncRNA16913.1 regulates FZD6 expression via competitively binding to chi-miR-195-5p. The findings of this study will offer new insights into the ceRNA-dependent mechanism underlying melatonin-governed cashmere growth and lay a theoretical foundation for the genetic improvement of cashmere goats.

## 2. Materials and Methods

### 2.1. Cell Culture

Primary skin fibroblasts of Liaoning cashmere goats used in this study were preserved in our laboratory. These cells were isolated from the scapular skin tissues of six 3-month-old male Liaoning cashmere goats using the tissue explant adherent method. Cells were cultured in a constant-temperature incubator (Shanghai Lixin Instrument, Shanghai, China) at 37 °C with 5% CO_2_. After the initial 24 h of culture, the medium was replaced with fresh complete medium. The complete medium was prepared by mixing high-glucose DMEM (KGL1206-500, KeyGEN Bio, Nanjing, China) and fetal bovine serum (BMC1020, Yakeyin Biotechnology, Wuhan, China) at a volume ratio of 9:1. Skin fibroblasts at passage 3 were used for all subsequent experiments. For melatonin treatment, cells were incubated with 200 ng/L melatonin (M252590, Sigma, St. Louis, MO, USA) dissolved in DMSO (D8371, Solarbio, Beijing, China), while the control group was treated with an equal volume of DMSO solvent only; all cells were harvested after continuous 48 h incubation.

### 2.2. Immunohistochemistry

Skin tissues were collected from three healthy 3-month-old male Liaoning cashmere goats for section preparation. Three biological replicates were set, and a total of nine valid sections were obtained. Sections were subjected to citrate antigen retrieval, followed by incubation with 3%H_2_O_2_ at 37 °C for 15 min to block endogenous peroxidase activity. All sections were rinsed three times with PBS for 5 min per wash. After removing residual liquid, sections were blocked with 5% BSA at 37 °C for 1 h and thoroughly washed with PBS. Diluted FZD6 pAb (A10503, Abclonal, Wuhan, China, 1:50) was applied to the experimental samples. PBS was added to the negative control group. All sections were incubated overnight at 4 °C in a humidified chamber. After rewarming to room temperature for 1 h and PBS washing, sections were incubated with secondary antibody (HRP Goat Anti-Rabbit IgG, AS014, Abclonal, Wuhan, China, 1:50) at 37 °C for 30 min, followed by another round of PBS rinsing. Freshly prepared 3,3′-diaminobenzidine (DAB) working solution was added for color development, and this was monitored under a microscope and terminated with PBS. Nuclei were counterstained with hematoxylin for 5 min, and sections were rinsed with running tap water for 15 min. Differentiation was performed using 1% acetic acid for 1 s, followed by 15 min of rinsing and 7 min of blue restoration with ammonia water. Subsequently, sections were dehydrated with a graded ethanol series (100%, 95%, 90%, 80%, 70%, 50%) and cleared with xylene twice, for 3 min each. After mounting with neutral balsam and air-drying at room temperature, all sections were observed and imaged using an inverted microscope (BA310, Motic, Xiamen, China), and image analysis was conducted using Motic Image Plus 3.0 software.

### 2.3. Preparation of LncRNA16913.1 Overexpression Lentivirus

The full-length sequence of LncRNA16913.1 (GenBank submission: SUB16273371, detailed sequence listed in Table S1) was inserted into the shuttle vector pLVX-IRES-puro (BR025, Fenghui Biotechnology, Changsha, China) via *EcoR* I and *BamH* I restriction sites to construct the overexpression plasmid pLVX-LncRNA16913.1, with empty vector as control. The full recombinant pLVX-LncRNA16913.1 plasmid was subjected to Sanger sequencing to confirm complete sequence consistency with the target LncRNA16913.1 transcript before lentivirus packaging. Lentivirus was packaged in HEK-293T cells (iCell-h237, iCell Bioscience, Shanghai, China) using a three-plasmid system including pSPAX2 (BR036, Fenghui Biotechnology, Changsha, China) and pMD2.G (BR037, Fenghui Biotechnology, Changsha, China). Transfection was performed with Lipofectamine 3000. The culture medium was refreshed 6 h after transfection. Supernatants were harvested at 48 h and 72 h post-transfection; the two batches of supernatants collected at 48 h and 72 h were fully pooled prior to purification, filtered through a 0.45 μm filter, and concentrated by ultracentrifugation at 72,000× *g* for 120 min. After ultracentrifugation, the precipitated viral pellets were resuspended in serum-free DMEM medium. Viral aliquots were stored at −80 °C. Lentiviral titers of both overexpression and empty control viruses were quantified via RT-qPCR-based titer detection method, with titers determined as 1.3 × 10^8^ TU/mL and 2.2 × 10^8^ TU/mL, respectively. To eliminate the influence of unequal viral titers during cell infection, the empty control lentivirus was diluted with serum-free DMEM to match the titer of the LncRNA16913.1 overexpression lentivirus before cell transduction. Cashmere goat skin fibroblasts were infected with lentivirus at a fixed multiplicity of infection (MOI) of 30. Puromycin screening was not performed in this experiment, as puromycin selection was only applied for the construction of stable cell lines in our laboratory, and transient lentiviral transduction was adopted for this study without stable cell enrichment. Cells were collected 48 h after lentiviral infection, for subsequent analyses.

### 2.4. Cell Transfection

Cells were seeded in 24-well plates and cultured to approximately 30% confluence before transfection. A total of 100 nM of chi-miR-195-5p Mimics/Inhibitor, Mimics NC/Inhibitor NC and LncRNA16913.1 siRNAs were diluted with serum-free medium. Lipofectamine 3000 (L3000015, Invitrogen, Carlsbad, CA, USA) was diluted separately, mixed with the RNA diluents, and incubated at room temperature to form transfection complexes. The prepared complexes were added to each well containing 0.45 mL of complete medium. After 6 h of transfection, the cell growth status was monitored, and the cells were cultured for an additional 48 h for subsequent experiments.

Co-transfection was carried out in 6-well plates using Lipofectamine 3000 (L3000015, Invitrogen) in a 1 mL system. Briefly, 250 μL Opti-MEM was mixed with 5 μL Lipofectamine 3000 as Solution 1. Solution 2 was composed of 250 μL Opti-MEM, 2.5 μg plasmids, 5 μL 20 μM siRNA and 5 μL P3000. After mixing the two solutions and incubating for 15 min at room temperature, the mixture was added to cells. Cells were cultured at 37 °C with 5% CO_2_ and harvested for analysis at 48 h post-transfection.

chi-miR-195-5p Mimics/Inhibitor and LncRNA16913.1 siRNAs used in this experiment were provided by GenCefe Biotech (Wuxi, China). The sequences (5′-3′) are listed below: si-LncRNA16913.1-1: CCACCGAGUUAGAGGUUCATT; si-LncRNA16913.1-2: GGGAAGGAAGUCAGUCUCATT; si-LncRNA16913.1-3: CACCUCCUCUGCUCAAGA ATT. chi-miR-195-5p Mimics guide strand: UAGCAGCACAGAAAUGUUGG, Star strand: AACAUUUCUGUGCUGCUAUU; chi-miR-195-5p Inhibitor: CCAACAUUUCUGUGCUGCUA.

### 2.5. RT-qPCR

**Figure 1:**
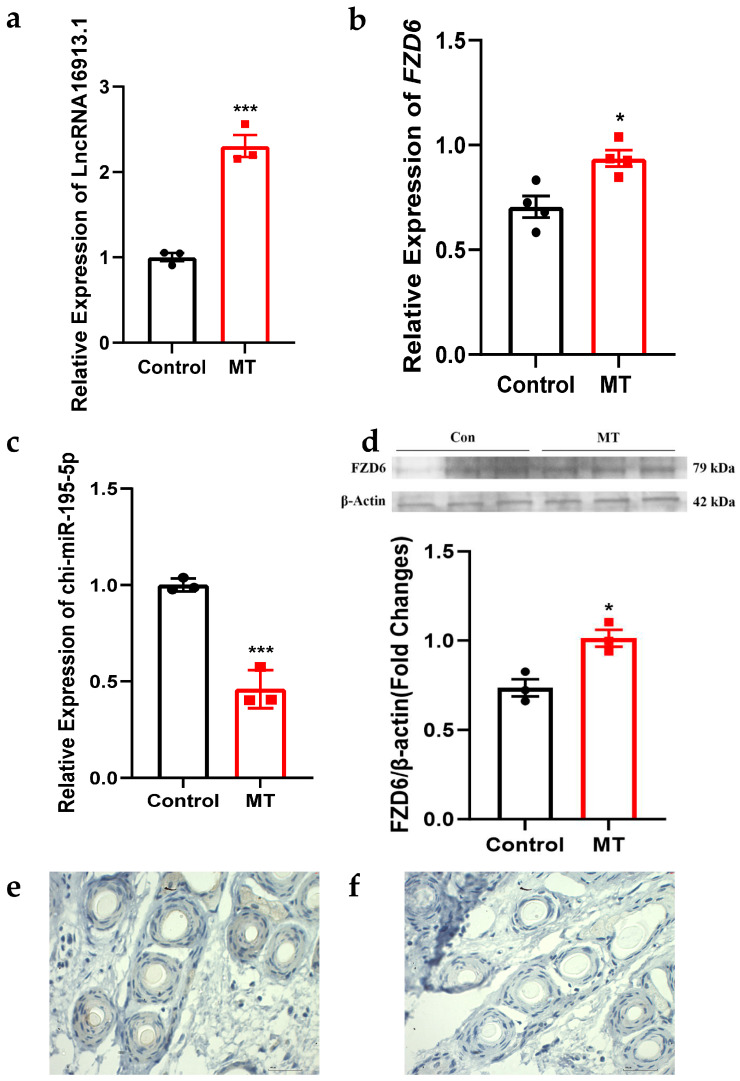
Regulation of lncRNA16913.1, chi-miR-195-5p and FZD6 expression by melatonin and localization of FZD6 in hair follicle tissues. (**a**–**c**): qPCR-detected relative expression of LncRNA 16913.1, chi-miR-195-5p and FZD6. Panel (**b**) (FZD6 qPCR): *n* = four independent biological replicates; panels (**a**,**c**): *n* = three independent biological replicates; (**d**) Western blot analysis of the regulatory effect of melatonin on FZD6 protein expression; (**e**) transverse localization of FZD6 in skin hair follicles (antibody group, 40× magnification); (**f**) negative control for FZD6 antibody (PBS, 40× magnification). All experiments were performed with three independent biological replicates, except Panel (**b**), and electrophoresis and Western blot were carried out in parallel, under consistent experimental conditions. * *p* ≤ 0.05, ***, *p* ≤ 0.001.

AG RNAex Pro RNA Reagent (AG21101, Accurate Biology, Changsha, China) was used to extract total cellular RNA according to the manufacturer’s instructions. Subsequently, 1 μg of total RNA was reverse-transcribed into cDNA using HP All-in-one qRT Master Mix (RT203, YoungGen, Kunming, China). RT-qPCR was performed with SYBR Green (SR4110, Solarbio, Beijing, China) to detect the expression levels of target genes, with GAPDH as the reference gene for data normalization. All data were measured utilizing the 2^−ΔΔCt^ method, with three replicate wells per sample. Most RT-qPCR gene quantification assays in this study utilized 3 independent biological replicates, while only the FZD6 mRNA detection under melatonin treatment (Figure 1b) adopted 4 independent biological replicates of Liaoning cashmere goat skin fibroblasts.

Primer sequences (5′ to 3′) used for qPCR are shown below: FZD6: Forward: AGAAGATTCAGATACCCAGAG, Reverse: CCACCGTGTCACCAAGT, the length of the amplified product is 148 bp; GAPDH: Forward: ATGTTTGTGATGGGCGTGAA, Reverse: GGCGTGGACAGTGGTCATAAGT, the length of the amplified product is 153 bp. LncRNA16913.1: Forward: GGGAAAACCACTACGAGACC, Reverse: ATGAGGGTACTCACAAGGTG, the length of the amplified product is 236 bp. chi-miR-195-5p Reverse transcription primer: CTCAACTGGTGTCGTGGAGTCGGCAATTCAGTTGAG CCAACATT, U6 Stem-loop Primer: GTCGTATCCAGTGCAGGGTCCGAGGTATTCGCACTGGATACGACCAAGCT, chi-miR-195-5p Forward: ACACTCCAGCTGGGTAGCAGCACAGAAA, Reverse: AGTGCAGGGTCCGAGGTATT, U6 Forward: CTCGCTTCGGCAGCACA, U6 Reverse: AACGCTTCACGAATTTGCGT.

### 2.6. Western Blot

Protein lysates were extracted from Liaoning cashmere goat skin fibroblasts using Wanleibio total protein lysis kit (WLA019, Wanleibio, Shenyang, China) supplemented with 1% PMSF protease inhibitor; cells were incubated on ice for 5 min, followed by centrifugation at 12,000 rpm, 4 °C, for 10 min, to collect supernatant protein solutions. The total protein concentration of each sample was quantified via BCA protein quantification kit (WLA004, Wanleibio, Shenyang, China) according to the manufacturer’s instructions, and equal amounts of 40 μg protein per sample were loaded for electrophoresis to guarantee consistent loading. Protein samples were denatured in boiling water for 5 min with 5× protein loading buffer before separation by 10% sodium dodecyl sulfate-polyacrylamide gel electrophoresis (SDS-PAGE). The 10% separation gel and 5% stacking gel were prepared using Wanleibio SDS-PAGE rapid gel preparation kit (WLA013, Wanleibio, Shenyang, China). Electrophoresis was performed at a constant voltage of 80 V for 2.5 h in 1× SDS-PAGE running buffer diluted from Wanleibio dry powder (WLA026, Wanleibio, Shenyang, China). The separated proteins were transferred onto 0.45 μm polyvinylidene fluoride (PVDF) membranes (IPVH00010, Millipore, Burlington, MA, USA); PVDF membranes were pre-activated with anhydrous methanol before transfer, and wet transfer was carried out at a constant voltage of 80 V for 1.5 h in pre-cooled transfer buffer composed of 3.03 g Tris, 14.4 g glycine, 200 mL methanol and ddH_2_O, to a final volume of 1 L. After transfer, the PVDF membranes were blocked with Wanleibio WB blocking solution (WLA066, Wanleibio, Shenyang, China) at room temperature with gentle shaking for 1 h, and then incubated with primary antibodies overnight at 4 °C, diluted by Wanleibio primary/secondary antibody dilution buffer (WLA068, Wanleibio, Shenyang, China). The primary antibodies and their dilution ratios were as follows: β-Actin mAb (AC038, Abclonal, Wuhan, China 1:10,000), FZD6 pAb (A10503, Abclonal, Wuhan, China, 1:5000). Subsequently, the membranes were washed three times (5 min per wash) with Wanleibio TBST washing buffer (WLA025, Wanleibio, Shenyang, China) and incubated with the corresponding secondary antibodies, HRP-conjugated goat anti-rabbit IgG (WLA023, Wanleibio, Shenyang, China, 1:5000 dilution), at room temperature for 1 h. After six rounds of TBST rinsing (5 min each), protein bands were visualized using Wanleibio ECL chemiluminescence substrate (WLA006, Wanleibio, Shenyang, China) and captured with a Tanon 5200 gel imaging system. Band gray-value quantification was completed with Tanon Image software (Version 2.03, Tanon, Shanghai, China) to calculate relative protein expression normalized to the internal reference β-actin for uniform protein-loading correction. All Western blot experiments were performed in three independent biological replicates.

### 2.7. CCK-8 Assay for Cell Viability Detection

Skin fibroblasts were seeded into 96-well plates at 1 × 10^5^ cells per well in 100 μL cell suspension. Three technical replicate wells were prepared for each treatment group, with blank control wells containing only complete culture medium (no cells) set up in parallel. At 12 h, 24 h, and 48 h after the interference, 10 μL of CCK-8 solution (BS350A, Biosharp, Hefei, China) was put into each well. The plates were gently mixed and incubated at 37 °C for 2 h. Next, the absorbance at 450 nm was observed. A microplate reader was used to measure optical density (OD) values of the experimental samples and the blank controls. The last corrected OD values were obtained by the formula: measured value—blank value.

### 2.8. Edu Assay for Cell Proliferation Detection

Skin fibroblasts were plated in 12-well culture dishes at a density of 2 × 10^5^ cells per well, and three separate biological duplicate batches were arranged for the EdU proliferation test. After siRNA transfection and 48 h continuous incubation, the original culture medium was discarded and replaced with fresh medium supplemented with EdU reagent, to reach a final working concentration of 10 μM. Cells were further incubated at 37 °C for 2 h, strictly following the operation guidelines of the Beyotime EdU staining kit (Cat. No. C0081S, Shanghai, China). Subsequently, cell fixation, membrane permeabilization and click-chemical staining steps were carried out sequentially, and DAPI dye was applied to stain all cell nuclei.

Fluorescence imaging was performed using a fluorescence microscope. A single representative visual field was photographed for every biological replicate, and all DAPI-labeled cell nuclei within each captured view were included for statistical counting. The ImageJ (Version 1.53k) analysis tool was adopted to quantify two types of cells separately: EdU-positive red fluorescence proliferating cells and total DAPI-stained cell nuclei in each field of view. The proliferation ratio of fibroblasts was calculated as the quotient of the number of EdU-positive cells divided by the total number of DAPI-stained nuclei from each captured microscopic view.

### 2.9. Cell Cycle Analysis by PI Single Staining

All flow cytometry assays were performed using a BeamCyte-1026 flow cytometer (BENM DIAG, Zhengzhou, China). A minimum of 10,000 cellular events were acquired for each individual sample during detection. Three independent biological replicates were conducted for all flow cytometry tests; each replicate represented an independently seeded and cultured fibroblast population for statistical analysis. Flow data were analyzed with FlowJo software (Version 10.10.1).

The cell cycle was analyzed using Cell Cycle Detection Kit (FXP021, 4A Biotech, Beijing, China). Harvested cells were centrifuged at 1000 rpm for 5 min, washed twice with pre-cooled PBS, and fixed overnight with ice-cold 70% ethanol, at 4 °C. After fixation, cells were washed twice with pre-cooled PBS to remove ethanol. Cell pellets were resuspended in 0.5 mL PI/RNase staining solution and incubated in the dark at room temperature for 30 min before flow cytometry detection.

Gating strategy and instrument parameters: in the FSC-A/SSC-A dot plot, the main cell population (P1) was gated to remove cell debris. FSC-W and SSC-W parameters were combined to exclude cell aggregates, and PI-A/PI-H dot plot was further used to remove adherent cell clusters. The single-cell population on the main diagonal was selected for DNA content analysis to distinguish cells at G0/G1, S and G2/M phases.

The instrument voltage was set as follows: FSC = 53 V, SSC = 7 V, PE = 50 V. The FSC threshold was set to 1 × 10^6^. Unstained blank control was used to adjust the negative cell population to the position of 10^3^ fluorescence intensity.

### 2.10. Apoptosis Detection by Annexin V-FITC/PI Double Staining

Cell apoptosis was detected using Annexin V-FITC/PI Apoptosis Detection Kit (FXP018, 4A Biotech). Cells were collected with trypsin without EDTA, resuspended in the collected culture medium, and centrifuged at 1000 rpm for 5 min. The supernatant was discarded, and cell pellets were washed twice with pre-cooled PBS. Cells were resuspended in 1 × binding buffer at a concentration of 1–5 × 10^6^ cells/mL. A total of 100 μL cell suspension was transferred to a flow tube, incubated with 5 μL Annexin V-FITC in the dark at room temperature for 5 min, and then stained with 10 μL propidium iodide (PI, 20 μg/mL). Finally, 400 μL PBS was added, and samples were immediately detected by flow cytometry.

Gating strategy and instrument parameters: cell populations were first gated in the FSC-A/SSC-A dot plot to exclude cell debris, dead cells and cell aggregates, and the main intact cell population (P1) was selected for subsequent analysis. A quadrant gating was set, based on unstained negative control cells, with the x-axis representing Annexin V-FITC fluorescence and the y-axis representing PI fluorescence. Cells in the lower-left quadrant (Annexin V^−^/PI^−^) were defined as viable cells; cells in the lower-right quadrant (Annexin V^+^/PI^−^) were early apoptotic cells; cells in the upper-right quadrant (Annexin V^+^/PI^+^) were late apoptotic or secondary necrotic cells; and cells in the upper-left quadrant (Annexin V^−^/PI^+^) were necrotic cells.

The instrument voltage was set as follows: FSC = 50 V, SSC = 20 V, FITC = 40 V, PE = 6 V. The FSC threshold was set to 1 × 10^5^. Unstained blank control was used to adjust the negative cell population to the position of 10^3^ fluorescence intensity.

### 2.11. Fluorescence in Situ Hybridization (FISH)

FISH assay was performed using GenePharma FISH Kit (Suzhou, GenePharma, China) on cells cultured on coverslips. Briefly, cell coverslips were fixed with 4% paraformaldehyde at room temperature for 10 min and washed three times with PBS (5 min each time). Membrane permeabilization was conducted with 0.5% Triton X-100 at 4 °C for 15 min, followed by another three PBS washes (5 min each). Subsequently, samples were incubated with 1 × blocking buffer at 37 °C for 30 min. After removing the blocking solution, samples were pre-treated with 2 × Buffer C at 37 °C, for 30 min. The probe powder was dissolved in DEPC-treated water to prepare a 100 μmol/L stock solution, which was further diluted to 1 μmol/L before use and denatured at 75 °C for 10 min. A total of 1 μL denatured probe was mixed with 1 μL SA-Cy3 and 8 μL PBS, and incubated at 37 °C for 30 min. The mixture was then blended with 90 μL pre-warmed Buffer E at 73 °C, and 100 μL probe hybridization mixture was added to each well for overnight hybridization at 37 °C in the dark. On the next day, the hybridization solution was discarded. Samples were washed with 0.1%Buffer F at 37 °C for 10 min, followed by three washes with 2 × Buffer C at 60 °C and another three washes at 37 °C (10 min each). Nuclei were stained with DAPI working solution in the dark for 5 min, and slides were rinsed twice with PBS (5 min each). After mounting with anti-fluorescence quenching reagent, images were observed and captured under a fluorescence microscope.

### 2.12. Bioinformatics Prediction of Binding Sites

The full-length sequence of chi-miR-195-5p was retrieved from the miRBase database. The online miRNA target prediction tool miRanda (https://cloud.oebiotech.cn/task/detail/array_miranda_plot/ (accessed on 15 May 2025)) was used to predict the binding sites between chi-miR-195-5p and the full-length sequence of LncRNA 16913.1. The prediction parameters were set as follows: the energy threshold was set to −10, and the score threshold was set to 120.

According to the goat reference genome (Capra hircus, ARS1.2) from NCBI, the wild-type 3′UTR sequences of FZD6 (XM_018058332.1) were obtained. The online miRNA target prediction tool miRanda (https://cloud.oebiotech.cn/task/detail/array_miranda_plot/ accessed on 15 May 2025) was applied to predict the binding sites between chi-miR-195-5p and the 3′UTR regions of FZD6. The screening parameters of miRanda were set as Energy < −10 and Score > 140.

### 2.13. Dual-Luciferase Reporter Assay

Wild-type and target mutant sequences were inserted into the pmirGLO vector by Sangon Biotech (Shanghai, China) to construct recombinant plasmids pmirGLO-LncRNA 16913.1-WT, pmirGLO-LncRNA 16913.1-MUT, pmirGLO-FZD6-WT and pmirGLO-FZD6-MUT.

Goat skin fibroblasts in the logarithmic growth phase were digested with 0.25% trypsin, diluted, and seeded into 24-well plates. Transfection was performed when the cell confluence reached 90%. Before transfection, the culture medium was replaced with 400 μL serum-free and antibiotic-free DMEM. In accordance with to the manufacturer’s instructions for the Lipo6000 transfection reagent (C0526, Beyotime), each well was co-transfected with 500 ng recombinant plasmid and 20 pmol miRNA using 2 μL transfection reagent. Six hours after transfection, the medium was replaced with complete medium, and cells were cultured for another 48 h.

The culture medium was discarded, and 200 μL dual-luciferase cell lysis buffer (RG132S, Beyotime) was added to each well for 10 min lysis on ice. The cell lysate was collected and centrifuged at 12,000 rpm for 3 min at 4 °C, and the supernatant was retained for subsequent detection. Working solutions were prepared following the instructions of the dual-luciferase reporter assay kit (RG088S, Beyotime). Briefly, 10 μL supernatant was transferred to a 96-well plate, followed by sequential addition of firefly luciferase and Renilla luciferase detection reagents. The luminescence intensity was measured using a Varioskan LUX multimode microplate reader (Thermo Fisher Scientific, Waltham, MA, USA).

### 2.14. RNA Pull-Down

Biotin-labeled RNA probes were used for RNA pull-down assay with a commercial kit (JKR23004, GeneKerry Biotechnology, Chengdu, China). Liaoning cashmere goat skin fibroblasts were collected and fully lysed on ice with lysis buffer, and an aliquot of the lysate was retained as the input control. Streptavidin magnetic beads were incubated with biotin-labeled target lncRNA probe or negative control probe at room temperature, for 2 h. After washing, the beads were mixed with cell lysate and incubated overnight at 4 °C to enrich endogenous RNAs interacting with lncRNA. Following thorough washing to remove non-specific binding components, the RNAs bound to the bead complexes were extracted using the Trizol method.

### 2.15. Data Analysis

Overall experimental design: three independent biological replicates were set for all cell-culture, transfection, drug-treatment, tissue-staining and molecular-detection experiments in this study. Each biological replicate consisted of independently seeded, treated and harvested cell populations, or independent goat tissue samples. All overexpression, knockdown, miRNA mimic/inhibitor and melatonin-intervention groups were equipped with corresponding negative control groups to eliminate vector-, RNA transfection- and solvent-interference factors. All rescue co-transfection groups were designed to mutually verify the upstream and downstream regulatory relationship of the ceRNA axis.

All data in this study were presented as mean ± standard deviation (SD). The Shapiro–Wilk test was used for normality verification, and all experimental data conformed to a normal distribution. One-way analysis of variance (one-way ANOVA) followed by Tukey’s post hoc multiple comparison test was applied to compare differences among multiple experimental groups. All in vitro cell experiments and tissue detection experiments were performed with three independent biological replicates, except for the FZD6 RT-qPCR quantification shown in Figure 1b, which was conducted with four independent biological replicates. The sample size and replicate number were determined according to the conventional standards of cellular- and molecular-biology experiments for livestock animals, which can ensure the reliability and repeatability of experimental results. A *p* value < 0.05 was considered statistically significant. Significance levels were denoted as follows: * *p* ≤ 0.05, ** *p* ≤ 0.01, *** *p* ≤ 0.001, and **** *p* ≤ 0.0001.

## 3. Results

### 3.1. Regulation of lncRNA16913.1, chi-miR-195-5p and FZD6 Expression by Melatonin and Localization of FZD6 in Hair Follicle Tissues

After Liaoning cashmere goat skin fibroblasts were treated with 200 ng/L melatonin for 48 h, the intracellular expression levels of LncRNA 16913.1, FZD6 and chi-miR-195-5p were detected. As shown in Figure 1a–c, melatonin treatment significantly increased the mRNA abundance of LncRNA 16913.1 (2.30-fold relative to the control group) and FZD6 (1.42-fold relative to the control group), while producing an extremely significant reduction in chi-miR-195-5p expression, which only reached 0.46 times the level of the control group. Western blot analysis further validated this regulatory pattern at the protein level: the relative protein content of FZD6 in the melatonin group was 1.38-fold that of the control group (Figure 1d).

Immunohistochemical staining was performed on transverse skin sections to detect the expression patterns of FZD6. The sections of negative control groups presented blue or pale-blue background staining (Figure 1e). In contrast, obvious tawny and sepia staining was observed in sections incubated with FZD6 antibodies, indicating specific immunoreactivity of FZD6 proteins. Further observation revealed that FZD6 was predominantly localized to the outer root sheath and inner root sheath of hair follicles (Figure 1f).

### 3.2. Effects of chi-miR-195-5p on the Proliferation of Liaoning Cashmere Goat Skin Fibroblasts

**Figure 2:**
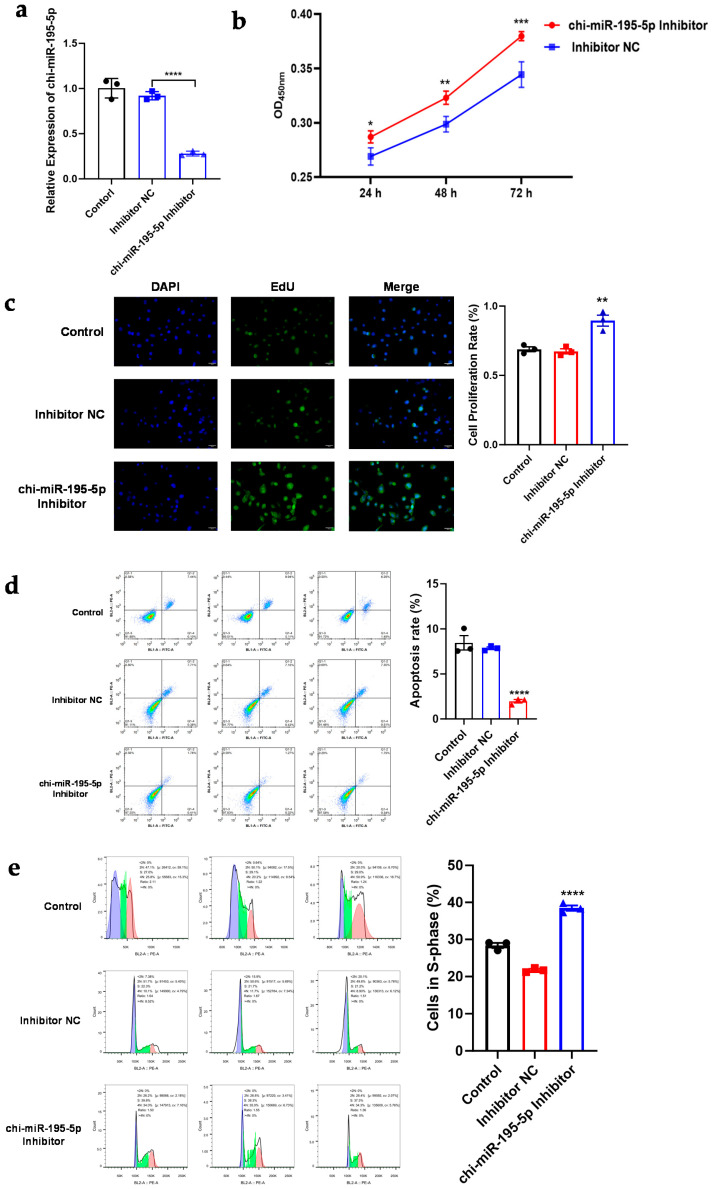
Effects of chi-miR-195-5p on the proliferation of Liaoning cashmere goat skin fibroblasts. (**a**) Interference efficiency of chi-miR-195-5p inhibitor detected by qPCR; (**b**) Cell viability measured by CCK-8 assay; (**c**) cell proliferation detected by EdU assay; (**d**) cell cycle distribution analyzed by flow cytometry; (**e**) cell apoptosis detected by flow cytometry. *, *p* ≤ 0.05; **, *p* ≤ 0.01; ***, *p* ≤ 0.001; ****, *p* ≤ 0.0001.

RT-qPCR was used to detect the interference efficiency of chi-miR-195-5p inhibitor. Figure 2a showed that transfection with chi-miR-195-5p inhibitor markedly downregulated the expression of chi-miR-195-5p, achieving an inhibition efficiency of 72.09% and confirming the validity of the inhibitor for subsequent experiments. CCK-8 results revealed that the viability of skin fibroblasts was significantly enhanced after chi-miR-195-5p knockdown (Figure 2b). EdU assays demonstrated that the cell proliferation rate in the inhibitor group (0.94% ± 0.08%) was obviously higher than that in the control group (0.78% ± 0.05%) (Figure 2c). Flow cytometry analysis showed the cell apoptosis rate in the inhibitor group was 1.97% ± 0.33%, which was notably lower than 7.85% ± 0.25% in the control group (Figure 2d). Meanwhile, the proportion of cells in S phase was significantly increased upon chi-miR-195-5p inhibition (Figure 2e).

### 3.3. chi-miR-195-5p Promotes Proliferation of Liaoning Cashmere Goat Skin Fibroblasts by Targeting FZD6

**Figure 3:**
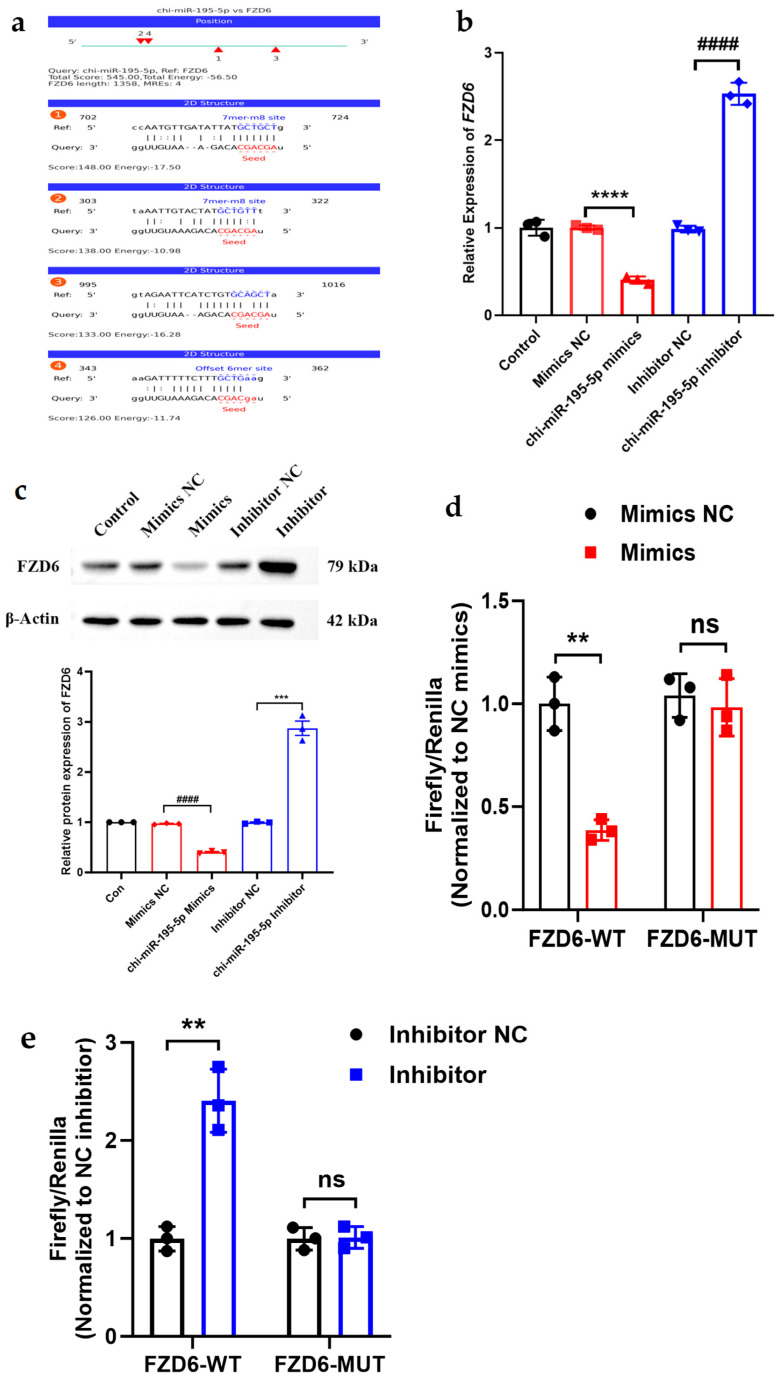
chi-miR-195-5p promotes the proliferation of skin fibroblasts from Liaoning cashmere goats by targeting FZD6. (**a**) Prediction of binding sites between chi-miR-195-5p and the 3′UTR region of FZD6; (**b**) qPCR detection of the negative regulation of FZD6 expression by chi-miR-195-5p; (**c**) Western blot detection of the negative regulation of FZD6 protein expression by chi-miR-195-5p; (**d**,**e**) dual-luciferase assay results. All samples came from parallel experiments, and electrophoresis and Western blot were carried out in parallel, under consistent experimental conditions. **, *p* ≤ 0.01; ***, *p* ≤ 0.001; ****, *p* ≤ 0.0001; ####, *p* ≤ 0.0001; ns, not significant.

The results showed that the seed sequence of chi-miR-195-5p (5′–AGCAGC–3′) bound to four regions within the 3′UTR of *FZD6* (XM_018058332.1), including 303–322 bp, 343–362 bp, 702–724 bp, and 995–1016 bp (Figure 3a). RT-qPCR and Western blot analyses demonstrated that transfection with chi-miR-195-5p inhibitor significantly elevated *FZD6* mRNA and protein levels: the relative *FZD6* mRNA expression reached 2.57–fold of the Inhibitor NC group, and the FZD6 protein abundance was 2.89–fold that of the Inhibitor NC group. In contrast, chi-miR-195-5p mimics remarkably suppressed FZD6 expression at both transcriptional and translational tiers, with *FZD6* mRNA reduced to only 40% and FZD6 protein decreased to merely 41% of the Mimics NC group, respectively (Figure 3b,c). These findings indicated a strong targeted interaction between chi-miR-195-5p and FZD6.

A dual-luciferase reporter assay was further performed to verify the targeting relationship between chi-miR-195-5p and FZD6. As shown in Figure 3d–e, in cells co-transfected with the wild-type recombinant plasmid pmirGLO-FZD6-WT, the luciferase activity was significantly increased in the chi-miR-195-5p inhibitor group compared with the inhibitor NC group. No significant difference in luciferase activity was observed between the two groups when cells were transfected with the mutant plasmid pmirGLO-FZD6-MUT. For cells transfected with pmirGLO-FZD6-WT, luciferase activity was obviously reduced in the chi-miR-195-5p mimics group relative to the mimics NC group. When pmirGLO-FZD6-MUT was applied, the luciferase activity showed no statistical difference between the chi-miR-195-5p mimics group and the mimics NC group. Collectively, these dual-luciferase results confirmed the direct binding of chi-miR-195-5p to the FZD6 3′UTR; rescue experiments further proved that altering FZD expression could completely reverse the proliferation, apoptosis and cell-cycle phenotypes induced by chi-miR-195-5p. These integrated molecular and cellular data strongly support the fact that FZD6 serves as the pivotal functional effector in this regulatory cascade in skin fibroblasts.

### 3.4. LncRNA 16913.1 Sponges chi-miR-195-5p in Liaoning Cashmere Goat Skin Fibroblasts

**Figure 4:**
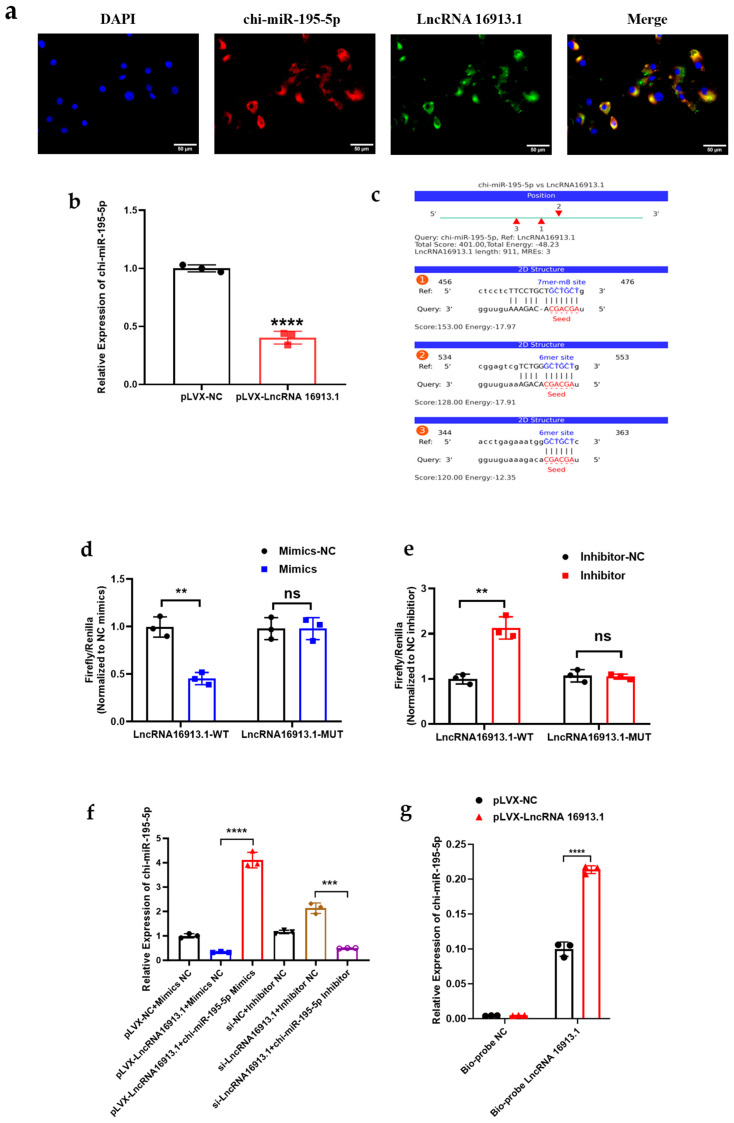
LncRNA16913.1 functions as a molecular sponge for chi-miR-195-5p in skin fibroblasts of Liaoning cashmere goats. (**a**) Subcellular localization of LncRNA 16913.1 and chi-miR-195-5p detected by fluorescence in situ hybridization (FISH); (**b**) relative expression of chi-miR-195-5p determined via qPCR; (**c**): bioinformatic prediction of binding sites between LncRNA16913.1 and chi-miR-195-5p; (**d**,**e**) dual-luciferase reporter assay verifying the targeted interaction between LncRNA16913.1 and chi-miR-195-5p; (**f**) rescue experiment of LncRNA16913.1 and chi-miR-195-5p, with chi-miR-195-5p expression quantified by qPCR; (**g**) RNA pull-down assay was conducted by incubating biotin-labeled LncRNA16913.1, its antisense RNA with cell lysates from the vector group and LncRNA16913.1 overexpression group. The enrichment level of chi-miR-195-5p was subsequently measured using RT-qPCR. **, *p* ≤ 0.01; ***, *p* ≤ 0.001; ****, *p* ≤ 0.0001; ns, not significant.

We first successfully constructed the overexpression vector pLVX-LncRNA16913.1, as validated in Figure S1. Three independent siRNAs targeting endogenous LncRNA16913.1 were designed and transfected into skin fibroblasts; si-LncRNA16913.1-1, which exhibited the highest knockdown efficiency, was selected for subsequent functional assays (Figure S2). FISH results demonstrated that both LncRNA 16913.1 and chi-miR-195-5p were predominantly distributed in the cytoplasm of skin fibroblasts (Figure 4a). RT-qPCR detection showed that overexpression of LncRNA 16913.1 significantly reduced the expression level of chi-miR-195-5p; the relative expression of chi-miR-195-5p in the LncRNA overexpression group was only 0.40-fold of that in the pLVX-NC control group (Figure 4b). Bioinformatic prediction identified three binding regions between the seed sequence 5′-AGCAGC-3′ of chi-miR-195-5p and LncRNA 16913.1, located at positions 344–363 bp, 456–476 bp and 534–553 bp (Figure 4c). Dual-luciferase reporter assay further confirmed the direct targeted binding between LncRNA 16913.1 and chi-miR-195-5p (Figure 4d,e). Bidirectional rescue experiments were performed to further clarify the regulatory interaction between LncRNA 16913.1 and chi-miR-195-5p. In the overexpression rescue assay, compared with the pLVX-NC + Mimic NC group, LncRNA 16913.1 overexpression markedly suppressed *FZD6* mRNA expression; when chi-miR-195-5p mimic was co-transfected together with LncRNA overexpression vector, this inhibitory effect was significantly reversed, and the FZD6 transcript level of cells co-transfected with LncRNA overexpression vector and chi-miR-195-5p mimic reached 12.33-fold that of cells only transfected with LncRNA overexpression vector and mimic negative control. In the knockdown rescue assay, LncRNA16913.1 silencing significantly upregulated *FZD6* mRNA expression relative to the si-NC+Inhibitor NC group, and this upregulation was abrogated by simultaneous transfection with chi-miR-195-5p inhibitor; the FZD6 expression level of cells co-transfected with LncRNA siRNA and chi-miR-195-5p inhibitor was reduced to only 0.23-fold of cells solely transfected with LncRNA siRNA and inhibitor negative control (Figure 4f). In vitro RNA pull-down assay validated the specific binding capacity of LncRNA16913.1 to chi-miR-195-5p (Figure 4g).

### 3.5. LncRNA16913.1 Upregulates FZD6 Expression via Sponging chi-miR-195-5p

**Figure 5:**
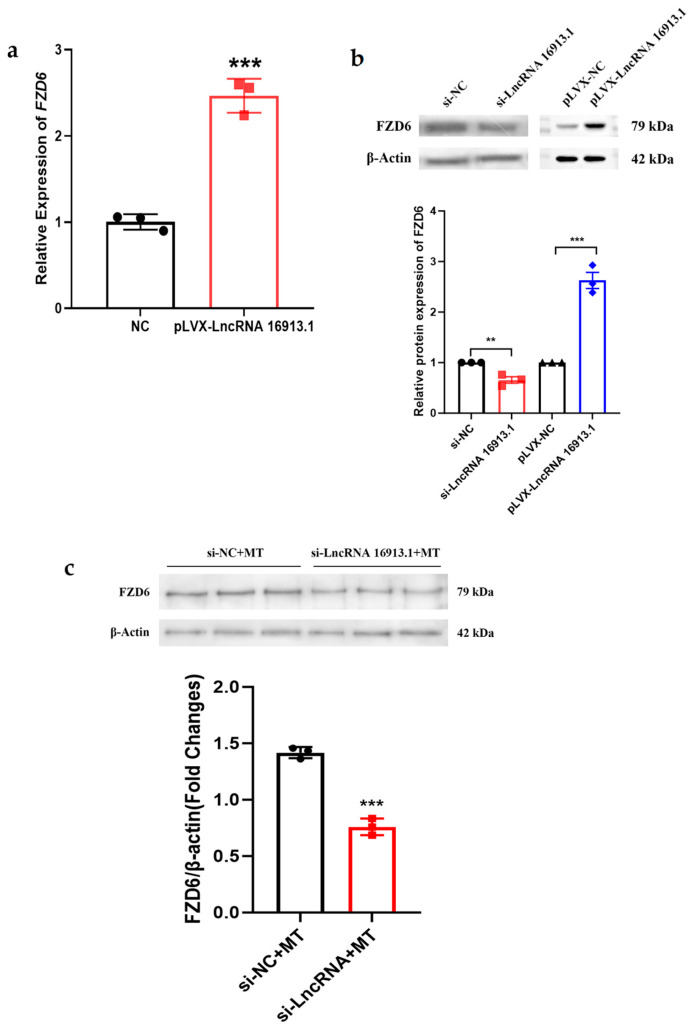

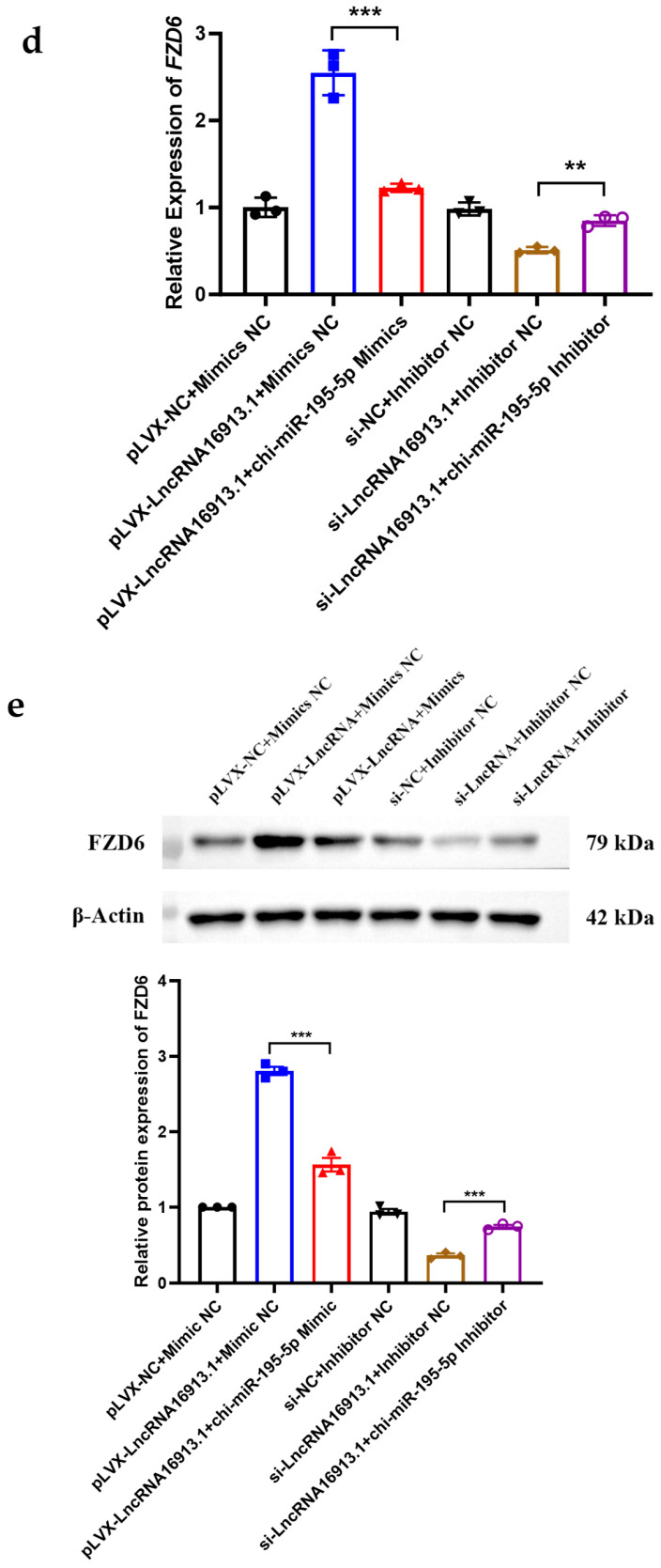
LncRNA16913.1 elevates FZD6 expression by sponging chi-miR-195-5p. (**a**) The mRNA expression level of *FZD6* detected by qPCR; (**b**,**c**) Western blot analysis of FZD6 protein expression; (**d**) rescue assay for LncRNA16913.1 and chi-miR-195-5p, qPCR was used to determine the mRNA level of *FZD6*; (**e**) rescue assay for LncRNA16913.1 and chi-miR-195-5p, Western blot was applied to measure FZD6 protein abundance. All samples came from parallel experiments, and electrophoresis and Western blot were carried out in parallel, under consistent experimental conditions. **, *p* ≤ 0.01; ***, *p* ≤ 0.001.

qPCR results revealed that overexpression of LncRNA16913.1 significantly increased the mRNA level of FZD6, with FZD6 transcript abundance in LncRNA-overexpressing cells reaching 2.46-fold that of cells transfected with empty vector control (Figure 5a). Consistent with the qPCR data, Western blot analysis demonstrated that LncRNA16913.1 overexpression markedly elevated the protein abundance of FZD6, to 2.63-fold of empty vector control, whereas knockdown of LncRNA16913.1 dramatically downregulated FZD6 protein expression, to only 0.65-fold of si-NC control (Figure 5b). Moreover, silencing LncRNA16913.1 reversed the promotive effect of melatonin on FZD6 protein expression; the FZD6 protein level in cells co-treated with LncRNA siRNA and melatonin was merely 0.54-fold of cells receiving si-NC plus melatonin treatment (Figure 5c).

Bidirectional rescue experiments were performed to further validate the fact that LncRNA16913.1 modulates FZD6 expression by sponging chi-miR-195-5p. In the overexpression rescue assay, LncRNA16913.1 overexpression significantly promoted *FZD6* mRNA expression relative to the pLVX-NC + Mimic NC group, and this facilitative effect was remarkably abolished by co-transfection with chi-miR-195-5p mimic, where the *FZD6* mRNA level of cells co-transfected with LncRNA overexpression vector and chi-miR-195-5p mimic decreased to 0.48-fold of cells only receiving LncRNA overexpression vector plus mimic negative control (Figure 5d). In the knockdown rescue assay, depletion of LncRNA16913.1 significantly suppressed *FZD6* mRNA expression compared with the si-NC + Inhibitor NC group, while co-transfection of chi-miR-195-5p inhibitor effectively rescued the inhibitory effect induced by LncRNA16913.1 knockdown, and *FZD6* mRNA expression of cells co-transfected with LncRNA siRNA and chi-miR-195-5p inhibitor recovered to 1.67-fold that of cells solely transfected with LncRNA siRNA and inhibitor negative control (Figure 5d). At the protein level (Figure 5e), co-transfection with chi-miR-195-5p mimic reduced FZD6 protein abundance to 0.56-fold of cells only overexpressing LncRNA, whereas simultaneous delivery of chi-miR-195-5p inhibitor restored FZD6 protein expression to 2.04-fold of cells only silencing LncRNA.

## 4. Discussion

Melatonin has become a research hotspot in the breeding and cashmere-yield improvement of Liaoning cashmere goats. Previous studies have verified the fact that melatonin activates the sulfur metabolic pathway by regulating the expression of genes related to cellular energy metabolism and the cell cycle, thereby supplying sufficient sulfur-containing amino acids for cashmere growth [21]. Subcutaneous implantation of melatonin in Liaoning cashmere goats during the non-cashmere-producing period significantly triggers the regeneration of secondary skin hair follicles, increasing the number of active follicles and cashmere yield, and this promotive effect exerts no adverse influences on subsequent hair growth cycles [22]. In addition, melatonin strengthens cellular antioxidant capacity and inhibits cell apoptosis, which is essential for maintaining the normal development of secondary hair follicles and improving the yield and quality of cashmere, and such beneficial effects persist throughout the lifespan of cashmere goats [23,24]. Other studies have demonstrated that pharmacological concentrations of melatonin modulate mitochondrial physiology and energy metabolism, thereby indirectly facilitating cell proliferation [25]. A melatonin concentration of 500 pg/mL is optimal for the proliferation of hair follicle stem cells derived from Inner Mongolian cashmere goats [26], while 1 μM melatonin can protect human dermal fibroblasts (HDFs) against UVB-induced damage [27]. Ge et al. [24] reported that melatonin may promote hair follicle elongation via lncRNA-mediated regulatory mechanisms.

Immunohistochemical results in this study revealed that FZD6 was mainly localized to the inner root sheath and outer root sheath of hair follicles. As an essential component of the hair follicle wall, the inner root sheath guides the normal growth of hair shafts, and its structural integrity is closely associated with hair follicle stability and fiber elongation [28]. The inner root sheath and hair cortex are two pivotal regions responsible for hair fiber elongation and structural formation [29]. The above expression patterns are highly consistent with the distribution characteristics of previously reported key regulatory factors in cashmere goat hair follicles, such as KRT14, FGF21 and Max [30,31,32], suggesting that FZD6 exerts important regulatory functions during hair follicle development and cashmere formation in Liaoning cashmere goats. Our previous transcriptomic data screened out melatonin-regulated LncRNA16913.1, chi-miR-195-5p and FZD6 [20]. The results of in vitro cellular experiments in the present study were consistent with the transcriptome sequencing data: melatonin treatment significantly upregulated the expression of LncRNA16913.1 and FZD6, whereas it downregulated chi-miR-195-5p, indicating that these three molecules participate in melatonin-mediated cellular regulation. In the present study, inhibition of chi-miR-195-5p dramatically promoted cell proliferation, accelerated cell cycle progression and suppressed apoptosis of Liaoning cashmere goat skin fibroblasts.

In recent years, lncRNA-mediated ceRNA regulatory networks have been proven to be extensively involved in diverse biological processes including hair follicle cycling, cell proliferation and apoptosis in cashmere goats, emerging as a core research field for elucidating the molecular mechanisms underlying cashmere growth [33,34,35]. The ceRNA mechanism works as follows: lncRNAs bind to miRNAs through sequence complementarity and thereby attenuate the inhibitory effects of miRNAs on their target genes. For instance, circRNA-1967 enhances the expression of LEF1 by sponging miR-93-3p, thus participating in the differentiation of cashmere goat secondary hair-follicle stem cells (SHF-SCs) toward the hair follicle lineage [36]. In addition, circRNA-0100 elevates KLF5 expression by sequestering miR-153-3p, consequently promoting the differentiation of secondary hair-follicle stem cells into the hair follicle lineage in cashmere goats [37].

FISH results in this study revealed that LncRNA16913.1 and chi-miR-195-5p were co-localized in the cytoplasm, which was consistent with the subcellular distribution characteristic whereby ceRNAs exert molecular sponge functions within the cytoplasm. Bioinformatic prediction identified multiple stable binding sites between the seed sequence of chi-miR-195-5p and LncRNA16913.1. Dual-luciferase reporter assay and RNA pull-down assay further verified the specific direct interaction between LncRNA16913.1 and chi-miR-195-5p at the in vitro molecular level. Gene expression detection demonstrated that overexpression of LncRNA16913.1 markedly reduced the abundance of chi-miR-195-5p, whereas knockdown of LncRNA16913.1 significantly restored chi-miR-195-5p expression. Furthermore, transfection of chi-miR-195-5p mimic or inhibitor completely reversed the regulatory effect of LncRNA16913.1 on this miRNA. Collectively, these findings are sufficient to demonstrate that LncRNA16913.1 acts as a ceRNA to specifically sponge chi-miR-195-5p in skin fibroblasts of Liaoning cashmere goats.

On the basis of clarifying the molecular interaction, this study further analyzed the regulatory effect of this ceRNA axis on the downstream functional gene *FZD6*. The qPCR and Western blot results showed that overexpression of LncRNA16913.1 significantly promoted the transcriptional and protein expression of FZD6, while silencing LncRNA16913.1 markedly inhibited FZD6 expression. Meanwhile, knockdown of LncRNA16913.1 effectively reversed the upregulatory effect of melatonin on FZD6, indicating that LncRNA16913.1 acts as an essential intermediate molecule for melatonin to modulate FZD6 expression. Bidirectional rescue experiments serve as a core strategy to verify ceRNA regulatory pathways. Our data revealed that chi-miR-195-5p mimic abolished the promotive effect of LncRNA16913.1 on FZD6, whereas chi-miR-195-5p inhibitor rescued the downregulation of FZD6 induced by LncRNA16913.1 knockdown. Combined with cellular phenotypic results, chi-miR-195-5p functions as a proliferation suppressor in skin fibroblasts of Liaoning cashmere goats. By sequestering and depleting chi-miR-195-5p, LncRNA16913.1 relieves the post-transcriptional repression of *FZD6*, and, ultimately, elevates its expression. Consistent with previous in vivo knockout evidence in mice that FZD6 controls hair follicle polarity and cyclic development, our in vitro data further complement the conserved function of FZD6 in cashmere goat skin cells. Although we did not conduct separate FZD6 overexpression/silencing tests in this work, all rescue phenotypic changes were fully dependent on FZD6 expression fluctuation, which solidified its central role downstream of chi-miR-195-5p.

There are several limitations to the present study. All experimental data were obtained from an in vitro monolayer skin fibroblast model, which cannot fully recapitulate the complex microenvironment featuring interactions among hair follicles, skin fibroblasts and stromal cells in intact animal skin tissue. Therefore, the expression pattern and biological function of the LncRNA16913.1-chi-miR-195-5p-FZD6 regulatory axis in skin tissues of living Liaoning cashmere goats, and hair follicles at distinct developmental stages and different physiological cycles remain to be further validated by subsequent in vivo experiments.

Limitations of the present study: this study only verified the ceRNA regulatory axis at the cellular and molecular level, and the causal relationship between this axis and cashmere-yield traits has not been confirmed in breeding cohorts. Subsequent in vivo injection experiments and population transcriptome association analyses will be conducted to provide in vivo evidence for this pathway.

## 5. Conclusions

In this study, we identified LncRNA16913.1 as a novel melatonin-responsive lncRNA, which functions as a molecular sponge to sequester chi-miR-195-5p and thereby upregulate the expression of FZD6. These findings elucidate a novel in vitro regulatory mechanism underlying melatonin-mediated regulation of cashmere goat skin fibroblasts, and offer candidate molecular targets for subsequent in vivo functional verification and follow-up research on cashmere goat genetic improvement.

## Data Availability

All data supporting the findings of this study are available within the article or Supplementary Materials. Further inquiries can be directed to the corresponding author.

## Supplementary Materials

The following supporting information can be downloaded at: https://www.mdpi.com/article/10.3390/ani16152420/s1, Figure S1: Detection of overexpression efficiency of pLVX-LncRNA16913.1 by qPCR; Figure S2: Detection of interference efficiency of LncRNA16913.1 by qPCR; Table S1: The full-length sequence of LncRNA 16913.1; File S1: Original Images.

## Abbreviations

The following abbreviations are used in this manuscript:

MT	Melatonin
ncRNA	Non-coding RNA
ceRNA	Competing endogenous RNA
FZD6	Frizzled 6
FISH	Fluorescence in situ hybridization
HDFs	Human dermal fibroblasts
SHF-SCs	Hair follicle stem cells
