# Supplementary material for: Melatonin Regulates the Proliferation of Liaoning Cashmere Goat Skin Fibroblasts via the lncRNA16913.1-chi-miR-195-5p-FZD6 Axis
Source: Animals (Basel). 2026 Aug 5;16(15):2420. doi: 10.3390/ani16152420 (PMC13465403; doi:10.3390/ani16152420)
Supplement: Supplementary file 1 [file animals-16-02420-s001.zip › animals-4420054-supplementary.pdf]

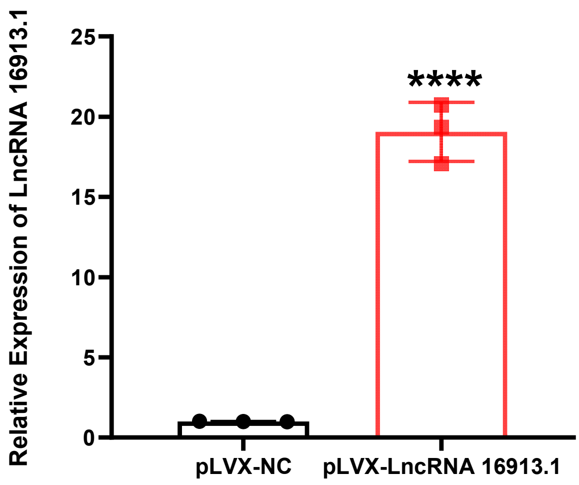

Figure S1: Detection of overexpression efficiency of pLVX-LncRNA16913.1 by qPCR.

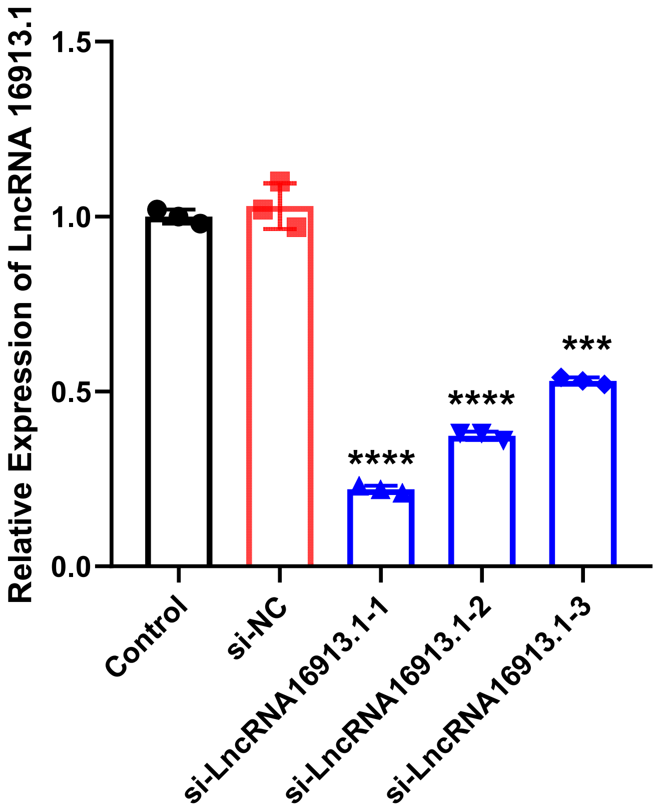

Figure S2: Detection of interference efficiency of LncRNA16913.1 by qPCR.
